# Non-Cohesive Gold Fillings

**Published:** 1910-05-15

**Authors:** J. V. Conzett

**Affiliations:** Dubuque, Ia.


### NON-COHESIVE GOLD FILLINGS.

J. V. CONZETT, D.D.S., DUBUQUE, IA.

Non-cohesive gold is more readily adapted to the walls of a cavity than any other filling material with which I am familiar, and when properly inserted makes a filling that is serviceable and permanent.

Since the publication of these papers a tooth was sent me by a friend in the East in which was a beautiful non-cohesive filling that had been doing service for over thirty years and was still as good as ever; and though the tooth had been lost, as a result of pyorrhea, the filling was still in place and was absolutely perfect. The use of non-cohesive gold calls for considerable skill and a knowledge of its physical characteristics. By reason of its non-cohesiveness its treatment must be entirely different from that of cohesive foil. One piece will not cohere to another, consequently each piece must be so placed in the cavity that it is mechanically locked in place. Obviously it cannot be used in all classes of cavities to the best advantage. It is true that the old dentists who practiced before the advent of cohesive gold used it in all classes of cavities, but in doing so they limited their ability to restore lost tooth tissue. With the non-cohesive foil it was impossible to build out a tooth to its original contour, consequently the fillings that were made in the approximal surfaces of the teeth were flat. While it is true that it is not indicated in all places, it is without a peer in its special province.

The cavities in which the non-cohesive foil is indicated either in whole or in part are cavities in the occlusal surfaces of bicuspids and molars, large, deep cavities in the gingival surfaces of the same teeth, the lingual pits of incisors and the gingival thirds of cavities in the approximo-occlusal surfaces of bicuspids and molars.

In preparing the non-cohesive foil for use, we take a sheet of gold and cut it into the desired size. If we wish to use a cylinder containing one-half of a sheet of gold, we cut the sheet in half; for a quarter in fourths, and so on. This piece of gold is now folded upon itself until it is in a ribbon shape the width we desire the cylinder to be, which should be about one-sixteenth of an inch longer than the depth of the cavity in which we wish to use it. The reason for this is that when placed in the cavity we wish the cylinder to reach from the floor of the cavity, and project about one-sixteenth of an inch beyond the cavity surface. After the ribbon of gold is folded to the proper width it is rolled into a cylinder by taking a good-sized Swiss broach, hat-pin or any other suitable instrument, rolling the ribbon around it loosely until it is all rolled upon iteslf, when it is of a cylindrical shape. The broach is now pulled out and with a pair of pliers the ends are gently squeezed together to cause the leaves of gold to be even in the cylinder, giving the cylinder a neat appearance. The sizes that are usually used are one-half sheet cylinders, one-quarter sheets and, in very rare instances, whole sheets. Occasionally we will find that cylinders of one-eighth sheets are very useful, especially in small cavities, such as those in the buccal pits of molars and lingual pits of incisors.

The method of using these cylinders in the various cavities is as follows: When making a filling in the occlusal surface of a bicuspid or molar, a cylinder is placed upon end against the distal wall of the cavity and with a large, foot-shaped plugger pressed to place. Another cylinder is placed against the side of the cavity and another one on the opposite side, when we will have in the distal surface of the cavity a wall of gold around three sides of the cavity, leaving an opening in between the cylinders; into this opening another cylinder is wedged in, the whole is malleted to place and the entire distal surface of the cavity is filled. The same process may now be repeated with the mesial surface. That is, a cylinder is laid against the mesial wall of the cavity and pressed to place, one against either lateral wall, and again we have a small space in between the cylinders that is unoccupied. The practice is, when filling entirely with non-cohesive foil, to wedge the cylinder against the lateral walls of the cavity with a strong wedge-shaped instrument, and as the instrument is rotated in the wedging process it leaves a triangular or conical space in the center. A cylinder is prepared to fit the space and then the whole filling is thoroughly malleted to place. The better way of doing this, however, is to finish this part of the filling with cohesive foil. After the cylinders have been placed all around the cavity and no more gold can be placed in that way, the central space can be filled with a mass of cohesive foil and then the whole occlusal surface heavily malleted to place, when you will have a filling that for adaptation and service cannot be beaten by any filling that it is possible for man to make. And not only have we a perfect filling, but the time that is consumed in its making is so small that the patient and dentist are both delighted.

Perhaps the best place to use the non-cohesive foil is in the gingival thirds of molars and bicuspids. It is fillings of this description that have called out the encomiums of dentists all over the country, as members of the Black Club have operated at clinics from time to time. And these cavities which were such "bugbears" to the large majority of the profession, that they were very largely filled with amalgam, were filled with ease and dispatch to the delight of the patient, especially those that had suffered the four and five hour sittings at the hands of the cohesive foil operators. And then these men after spending hours in making such fillings, would see them come back in a few years with failure at the gingival surface, due to their inability to perfectly adapt the cohesive foil to this surface. I know whereof I speak for it has been my fortune to have renewed hundreds of these fillings that have failed in just that way. But with the non-cohesive foil properly used in this position it is so different. In filling a cavity in the proximo-occlusal surface of a bicuspid, or molar, the principles are the same. A cylinder is placed in the linguo-gingival angle and pressed to place with a plugger; another cylinder is placed in the bucco-gingival angle and pressed to place. We now have both angles filled and a space between. Into this space another cylinder is inserted and pressed to place. With a large plugger point this mass of gold is malleted down, being careful that the line of force is always toward the center of the tooth and not away from it, for if so, your gold will be tipped out of the cavity. These cylinders must always be placed with the *ends* protruding from the cavity, never the *sides*, for if the sides were left exposed the leaves of gold would flake off, as we are dealing with gold that does not cohere. We now have the entire gingival portion of the cavity filled and a perfect adaptation of the gold to the gingival margin. That margin will never give us any more concern; it is filled for all time if the lines of the cavity have been carried out properly in our cavity preparation. The rest of this cavity will be filled with cohesive foil beginning at the occlusal step, building the gold over the non-cohesive foil in the gingival seat.

After the cohesive foil is in place we again come back to the malleting of the non-cohesive foil, for as yet there has been no lateral stress placed upon it and the cylinders are sticking out of the cavity.

A large foot plugger is now inserted into the proximal space and these cylinders are vigorously malleted into the cavity and our gingival margin is perfectly protected.

Cavities in the gingival surfaces of bicuspids and molars can be filled by the combined use of the non-cohesive and cohesive foils to very good advantage. In these cavities a cylinder is placed in the distal angle and lightly pressed to place, one in the mesial angle, and also pressed into position; then the space between can be filled with cohesive foil, carefully building the foil over the non-cohesive cylinders until they are locked into place, when the filling can be finished without trouble. It is rather difficult to keep the cylinders in place at first, and the beginner will find that he will probably have a number of failures before he becomes an adept at this class of operations.

The last class of cavities to be considered under this head is composed of those cavities occurring in the pits in the lingual surfaces of the incisors and the buccal surfaces of molars. These cavities are usually very small and can be readily and most expeditiously filled with one cylinder of the non-cohesive foil. A cylinder containing more than enough gold to fill the cavity is selected and drawn out into a conical shape, which makes a sort of a cornucopia of the cylinder.

Into the cavity the small end of the cone is placed and with a large plugger the rest of the cylinder is forced into the cavity. If properly done the leaves of gold will slide over each other as they follow the point of the cylinder into the cavity, and as more pressure is applied the whole cylinder is forced into the cavity, when the mallet is brought into play and the whole filling thoroughly condensed.

Some operators prefer to reverse the method of placing the cone, and place the base into the cavity first. This always seemed wrong to me because of the difficulty of inserting a mass of gold that was as large as such a cylinder must be. I never had any success in doing it in that way, so devised the other way which is so much more simple and, to me at least, very much easier.

In case the cavities are too large to justify the filling of them with one cylinder, the same method can be applied to them that we apply to the occlusal cavities in molars and bicuspids, viz: cylinders can be placed around the cavity and in this way the center filled in with the cohesive foil.

Results as good as the above can be obtained by using ribbons of non-cohesive foil instead of the cylinders in some cases. For instance, in shallow cavities in the approximal surfaces of bicuspids and molars, where a great mass of non-cohesive foil is not desirable, the ribbon of foil can be folded upon itself until it assumes the form of a mat a little longer than the cavity is bucco-lingually, and this mat can be placed upon the floor of the gingival seat, pressed to place and the cohesive foil built over it as if it were a mass of malleted cylinders. Again in small box cavities a ribbon of non-cohesive foil can be laid all around the sides of the cavity and the center filled with cohesive foil. Either of these methods will give equally good results when carried out with the proper technic. The use of non-cohesive foil does not demand a large amount of skill. It does, like anything else, demand a certain amount of patience and perseverance; but when the ability to use it is acquired, the results obtained will more than repay the time spent in acquiring the necessary technic.—*Dental Digest.*

